# Commentary: Triggering Receptor Expressed on Myeloid Cells-1 Inhibitor Targeted to Endothelium Decreases Cell Activation

**DOI:** 10.3389/fimmu.2020.00173

**Published:** 2020-02-11

**Authors:** Alexander B. Sigalov

**Affiliations:** SignaBlok, Inc., Shrewsbury, MA, United States

**Keywords:** TREM-1, sepsis, SCHOOL model of receptor signaling, ligand-independent inhibition, TREM-1 inhibitory SCHOOL peptides

Triggering receptor expressed on myeloid cells-1 (TREM-1), an inflammation amplifier, first reported in 2000 (1) was initially demonstrated to play a role in sepsis (2). Currently, the crucial pathophysiological role of TREM-1 is defined not only in infectious diseases but also in both acute and chronic forms of aseptic inflammation (3) as well as in different types of cancer (4, 5). Examples are ischemia-reperfusion, hemorrhagic shock, pancreatitis, spinal cord injury, inflammatory bowel diseases, rheumatic diseases, retinopathy, liver diseases, atherosclerosis, psoriasis, cystic fibrosis, Parkinson's disease, lung cancer, pancreatic cancer, liver cancer, and colon cancer. This implicates TREM-1 as a new, highly promising multi-indication therapeutic target.

Conventional TREM-1 inhibitors such as either TREM-1 inhibitory peptides LP17 and LR12 first reported in 2006 (6) and 2013 (7), respectively, or an antibody against TREM-1 first reported in 2016 (8), all attempt to block the receptor binding to its ligand(s). However, the actual nature of the TREM-1 ligand(s) is still uncertain, emphasizing the hurdles that need to be overcome before TREM-1-targeted therapy can become a clinical reality.

To address this problem, we applied our model of receptor-mediated transmembrane signaling, the Signaling Chain HOmoOLigomerization (SCHOOL), first published in 2004 (9, 10) to rationally design TREM-1-specific inhibitory peptide sequence(s) (SCHOOL peptides/sequences) that employ a novel, ligand-independent mechanism of TREM-1 inhibition. We then successfully demonstrated high efficacy of these peptides/sequences in a variety of *in vitro* and *in vivo* studies (Table 1). Recently, therapeutic efficacy of the SCHOOL peptides/sequences has been independently confirmed in a mouse model of liver cancer [(15); Table 1].

**Table 1:** Published *in vitro* and *in vivo* studies of TREM-1 inhibitory peptide sequences that employ a ligand-independent molecular mechanism of TREM-1 inhibition (SCHOOL inhibitors).

**Sequence**	Origin^*^	In vitro studies^**^	**Animal model**	**Year**	**References**
GLLS**K**SLVF	mTREM-1_210−218_	LPS-stimulated cells	Sepsis Non-small cell lung cancer	2014	(11)
GFLS**K**SLVF	hTREM-1_213−221_	–	Collagen-induced arthritis	2017	(12)
GFLS**K**SLVF	hTREM-1_213−221_	–	Pancreatic cancer	2017	(4)
GFLS**K**SLVF	hTREM-1_213−221_	–	Oxygen-induced retinopathy	2018	(13)
GFLS**K**SLVF	hTREM-1_213−221_	–	Alcohol-induced liver disease	2019	(14)
GFLS**K**SLVF	hTREM-1_213−221_	–	Liver cancer	2019	(15)
LS**K**SLVF	hTREM-1_215−221_	LPS-stimulated cells	Sepsis	2019	(16)

* *mTREM-1, mouse TREM-1; hTREM-1, human TREM-1*.

** *LPS, lipopolysaccharide*.

The recently published manuscript by Gibot et al. (16) describes the use of ligand-independent modulation of TREM-1 to reduce lipopolysaccharide (LPS)-induced cell activation and confer protection during experimental sepsis in mice. To inhibit TREM-1 in a ligand-independent manner, the authors used a peptide sequence LSKSLVF (Table 1), which they claimed they rationally designed.

In this regard, we thought it proper to remind the readership of Frontiers in Immunology of our pioneering study of 2014 that demonstrated the therapeutic effect of a first-in-class ligand-independent TREM-1 inhibitory peptide sequence GLLSKSLVF (mouse TREM-1-specific

SCHOOL peptide) in experimental sepsis [(11); Table 1]. In this study, we presented for the first time, direct evidences that GLLSKSLVF suppresses TREM-1-mediated production of proinflammatory cytokines TNF-α, IL-1β, and IL-6 both *in vitro* (LPS-stimulated cells) and *in vivo* (LPS-challenged mice) as well as significantly prolongs survival of mice with LPS-induced septic shock (11). We specifically demonstrated that a control peptide (GLLSGSLVF) with single amino acid substitution of functionally important lysine (highlighted in bold type in Table 1) for glycine does not exhibit TREM-1 inhibitory effect, as it has been predicted by the SCHOOL model (10, 11).

We note that the paper by Gibot et al. does not refer to this previously published work (11). It should be also noted that in our another study, one of the cancer studies cited [Shen and Sigalov (4) in the paper by Gibot et al. (16)], we used a ligand-independent human TREM-1 inhibitory peptide GFLSKSLVF (GF9), not peptide LR12, to suppress tumor growth and prolong survival of mice with experimental pancreatic cancer (4).

In summary, we believe that it is important that our novel and clinically relevant ligand-independent approach to modulation of diverse immune receptors including TREM-1 [recently reviewed in Sigalov (17)] attracts more and more attention from the scientific and industrial community.

## Author Contributions

AS conceived and wrote the manuscript.

### Conflict of Interest

AS is employed by SignaBlok, Inc., a company developing ligand-independent TREM-1 inhibitors.
